# Comparative short-term and long-term outcomes between internal and external intestinal plication in the management of small bowel obstruction

**DOI:** 10.1186/s12893-021-01304-1

**Published:** 2021-07-12

**Authors:** Song Liu, Qiongyuan Hu, Lihua Shao, Xiaofeng Lu, Xiaofei Shen, Shichao Ai, Ping Zeng, Meng Wang, Wenxian Guan

**Affiliations:** 1 Department of Gastrointestinal Surgery, Nanjing Drum Tower Hospital, 321 Zhongshan RD, Nanjing, China; 2 Medical School of Nanjing University, Nanjing, China; 3 Nanjing Medical University, Nanjing, China; 4 Faculty of Hepato-Pancreato-Biliary Surgery, Chinese PLA General Hospital, Beijing, China

**Keywords:** Plication, Splinting, Stent, Bowel obstruction, Cocoon, Volvulus, Intussusception

## Abstract

**Background:**

Small bowel obstruction (SBO) is common and usually requires surgical intervention. Intestinal plication is a traditional but critical strategy for SBO in certain scenarios. This study is to compare the short-term and long-term outcome between internal and external plications in the management of SBO.

**Methods:**

All patients receiving intestinal plication in our hospital were retrospectively collected. Short-term outcome including postoperative complications, reoperation, postoperative ICU stay, starting day of liquid diet and postoperative hospitalization, as well as long-term outcome including recurrence of obstruction, readmission, reoperation and death were compared between groups. Gut function at annual follow-up visits was evaluated as well.

**Results:**

Nine internal and 11 external candidates were recruited into each group. The major causes of plication were adhesive obstruction, abdominal cocoon, volvulus and intussusception. Lower incidence of postoperative complication (*p* = 0.043) and shorter postoperative hospitalization (*p* = 0.049) was observed in internal group. One patient receiving external plication died from anastomosis leakage. During the 5-year follow-up period, the readmission rate was low in both groups (22.2 % vs. 9.1 %), and none of patients required reoperation or deceased. None of patients exhibited gut dysfunction, and all patients restored normal gut function after 4 years. Patients in external group demonstrated accelerated recovery of gut function after surgery.

**Conclusions:**

This study compares short-term and long-term outcome of patients receiving internal or external intestinal plication. We suggest a conservative attitude toward external plication strategy. Surgical indication for intestinal plication is critical and awaits future investigations.

**Supplementary Information:**

The online version contains supplementary material available at 10.1186/s12893-021-01304-1.

## Background

Small bowel obstruction (SBO) is a common gastrointestinal disease. Although conservative management such as nasogastric decompression plus somatostatin could be effective for most cases, surgical intervention is still critical in certain scenarios especially for ischemic, strangulated or refractory obstructions. Delay in surgical management could result in high risk of mortality and morbidity.

Intestinal plication is a traditional surgical technique that is suggested in the management of chronic, idiopathic or high risk of recurrent SBO. Intestinal plication carries considerable damages to the digestive and other systems, and leaves rare chance of re-laparotomy. Therefore, it is considered as the last and prudent strategy for SBO [1].

According to the different principles of bowel sequence and fixation, intestinal plication could be categorized into two major types, i.e., the internal plication and the external plication. Internal intestinal plication was also named intestinal tube splinting, intestinal stenting or sutureless plication [2, 3]. Its fundamental principle is to place a sustaining and guiding tube into the intestinal lumen. The elasticity and stiffiness of intraluminal tubes could ensure the correct sequence and sufficient curve of small bowel.

External intestinal plication was initially invented by Nobel and was therefore named Noble procedure [4]. Noble procedure indicated an organized bowel plication followed by continuous catgut suturing from the mesenteric root to the mesenteric margin and then along the mesenteric margin between adjacent bowels. This technique was modified by Childs in 1960 [5]. Since then, technical development was emerging to improve the safety and effectiveness of external intestinal plication [6].

Understanding the safety, effectiveness and long-term outcome of intestinal plication is necessary for surgeons. Although several previous studies have shared their clinical experiences [7, 8], neither comparison between internal and external plication nor long-term postoperative recovery of gut function was reported in literature.

In this study, we aim to compare the safety and effectiveness between internal and external intestinal plication in the management of SBO caused by a specific spectrum of diseases. Moreover, we will summarize the long-term outcome, especially the 5-year surveillance of gut function recovery after surgery in these patients.

## Materials and methods

### Patient recruitment and data collection

All patients that were registered at our database were retrospectively screened. The database was technically supported by *Yidu Cloud, Inc.* that automatically collected and integrated raw data of all patients during hospitalization and follow-up visits. The inclusion criteria were (1) definitive diagnosis of SBO, (2) surgical procedure of either internal or external intestinal plication, (3) data integrity during perioperative and long-term follow-up periods.

The diagnosis of small bowel obstruction was based on the clinical symptom, physical examination, laboratory test and especially abdominal CT scan [9]. All patients with suspicious bowel obstruction would receive a routine abdominal CT after admission in our hospital. An emergent intestinal plication was defined as the surgical procedure performed within initial 24 h after admission. The decision of either emergent or elective intestinal plication was made by the attending surgeon based on the clinical manifestation, abdominal examination as well as type, site and severity of obstruction.

Indication for intestinal plication mainly included (1).

Short-term outcome was defined as data collected from operation to discharge, and included postoperative complications (e.g., bleeding, leakage and infection), reoperation due to complications, postoperative ICU stay, starting day of liquid diet and duration of postoperative hospitalization.

Long-term outcome was defined as data collected at each visit after discharge, and included recurrence of bowel obstruction, readmission and reoperation due to bowel obstruction, and death during follow-up period. The postoperative gut function was evaluated at each annual visit using I-FEED scoring system [10]. I-FEED abbreviates for “intake, feeling nauseated, emesis, physical exam, and duration of symptoms”, and is designed by the American Society for Perioperative Quality Initiative that has taken into account of clinical signs, symptoms and implications. Gut function is categorized into normal (0–2 points), intolerance (3–5 points) and dysfunction (> 5 points) according to the I-FEED score.

### Surgical procedure

**Fig. 1:**
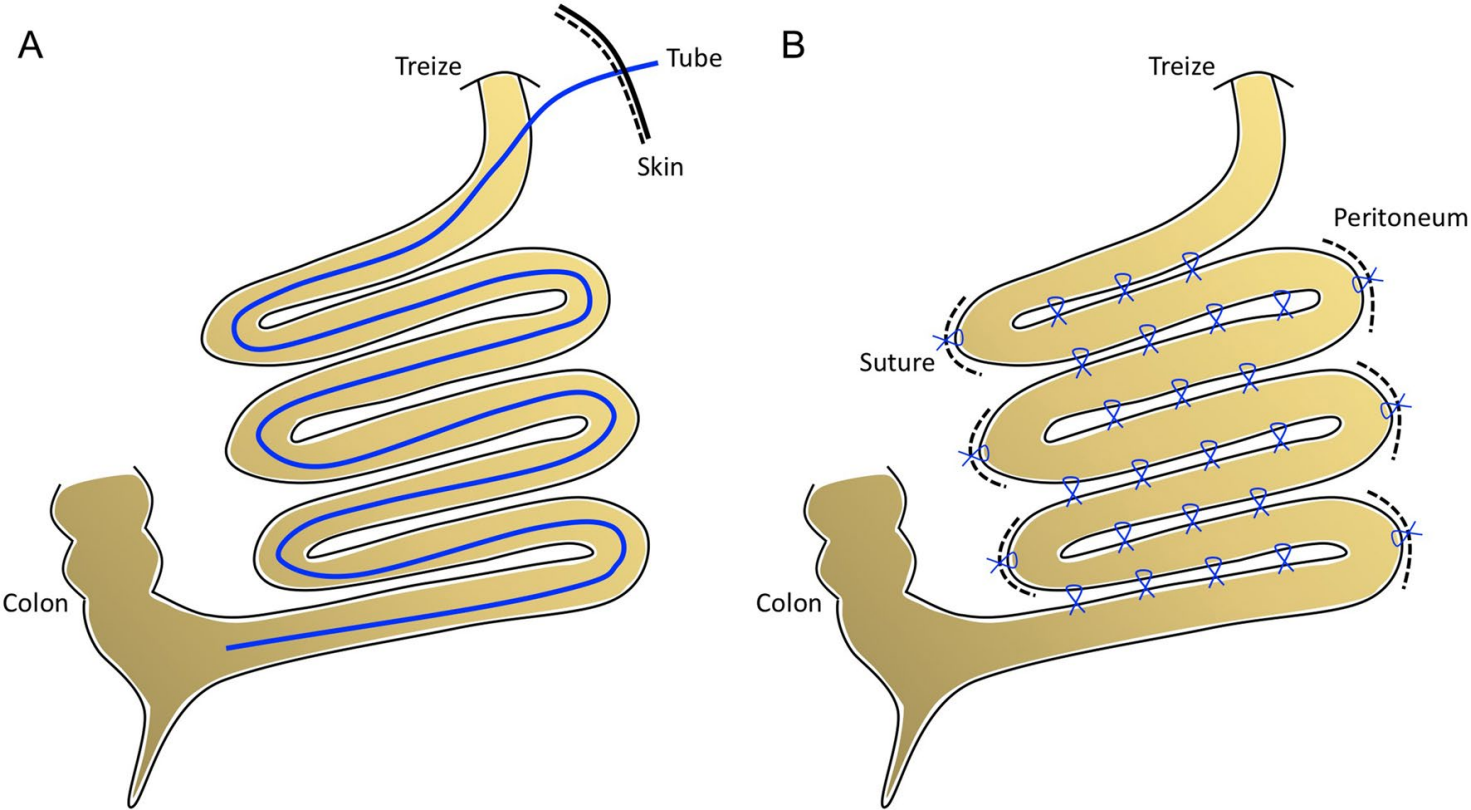
Graphic illustration of internal and external intestinal plication surgery. **A** Internal intestinal plication (also named intestinal tube splinting, intestinal stenting or sutureless plication). **B** External intestinal plication (modification of Nobel-Childs procedure)

The intestinal plication was performed as the following steps (Fig. 1). (1) restore intussusception or volvulus, or dissociate adherent bowels if the primary disease was abdominal cocoon or adhesive obstruction. (2) resect partial bowel segment where extensive inflammation or serosa defect existed. (3) plicate the jejunum and ileum into continuous loop-like shape to bring the adjacent bowels into apposition, in which step sharp corner angle should be avoided. (4) for internal plication, connect several F16 nasogastric tubes by end-to-end, put it into the jejunal lumen approxiamately 20 cm distal of Treize ligament, and then extend the tube until the terminal ileum. (5) for external plication, perform interrupted Lembert seromuscular Vicryl suture at antimesenteric boarder of adjacent bowels, and then perform interrupted Vicryl suture between seromuscular layer of bowel at turning corner and adjacent peritoneum. The purpose of small bowel plication is to induce controlled and predictable adhesions to prevent adhesive obstruction. All procedures were performed by a same surgical team including 2 senior attendings, 2 attendings and 3 residencies.

### Statistical analysis

All categorical variables were described as frequency and percentage. All continuous variables were presented as median (IQR). Chi-test was used for comparison of categorical variables, and fisher exact test was additionally utilized where applicable. Unpaired t-test was used for comparison of continuous variables, and Welch’s correction was additionally adopted where applicable. Statistical difference was defined as *p* < 0.05. All analysis and relevant figures were performed in GraphPad 7.0a for Mac OS X.

### Ethics

This study has been approved by the Ethics Committee of Nanjing Drum Tower Hospital (DTH-2020-IRB-031B, Dec. 15th, 2020). Consent wavier has approved by the Ethics Committee at Nanjing Drum Tower Hospital. All methods were carried out in accordance with relevant guidelines and regulations.

## Results

### Demographic and clinical features

Between 2010 and 2020, a total of 20 patients that received intestinal plications were enrolled into this study (Additional file 1:  Table S1). Among these 20 patients, 9 were allocated into internal group while the other 11 were assigned into external group. Table 1 demonstrated clinical features of the two groups. Male predominance was observed in both groups, with a proportion of 77.8 and 63.6 %, respectively. Middle-aged distribution was observed in both groups as well, with a mean age of 44.3 and 58.5, respectively. Similar BMI was found between groups (20.3 vs. 21.3 kg/m^2^).

**Table 1:** Clinical features between patients receiving internal or external intestinal plication

	Internal intestinal plication (n = 9)	External intestinal plication (n = 11)	*p*
Male (n, %)	7 (77.8 %)	7 (63.6 %)	0.64
Age (years)	36.0 (28.0)	60.0 (19.5)	0.33
BMI (kg/m^2^)	20.0 (1.6)	21.3 (2.7)	0.22
Primary disease (n, %)			0.63
Volvulus	3 (33.3 %)	2 (18.2 %)	–
Intussusception	0	1 (9.1 %)	–
Abdominal cocoon	1 (11.1 %)	4 (36.4 %)	–
Adhesive obstruction	5 (55.6 %)	4 (36.4 %)	–
Past abdominal surgery history (n, %)	8 (88.9 %)	7 (63.6 %)	0.32
Concomitant disease (n, %)	1 (11.1 %)	2 (18.2 %)	0.99
Pre-op WBC (×10^9^/L)	5.1 (3.0)	6.1 (2.1)	0.81
Pre-op hemoglobin (g/L)	112.0 (17.0)	126.0 (15.0)	0.039
Pre-op albumin (g/L)	35.5 (9.7)	36.2 (4.2)	0.83
Pre-op CRP (mg/dL)	4.1 (57.8)	6.8 (33.0)	0.91

The primary causes of intestinal plication included adhesive obstruction, abdominal cocoon (also named idiopathic sclerosing peritonitis), volvulus and intussusception, among which adhesive obstruction was the most frequent type of primary diseases for patients in both groups (55.6 % vs. 36.4 %). Statistical difference in the constitution of primary causes was not found between groups. The majority of patients suffered from past abdominal surgery in both groups (88.9 % vs. 63.6 %), and all patients that suffered from adhesive obstruction exhibited past abdominal surgery history.

Preoperative WBC and albumin were relatively normal in both groups, and were statistically similar between groups. Preoperative C-reactive protein was slightly higher than normal range but was similar between groups. Notably, patients that received external plication exhibited higher hemoglobin compared to those that received internal intestinal plication before surgery (128.6 vs. 115.0 g/L, *p* = 0.039).

### Surgical procedure

Approximately one third of intestinal plications were emergent surgery (33.3 % vs. 36.4 %) (Table 2). The mean duration of surgery was 263.6 and 313.6 min for internal and external plications, respectively. Intraoperative blood lost was slightly more in external plication (545.5 ml) compared to that in internal plication (183.3 ml). Intraoperative transfusion was not required except for 1 patient in external group. Partial small bowel resection was performed in 66.6 % patients in internal group compared to 45.5 % patients in external group. The mean resected segment of small bowel was 30.7 and 25.0 cm in internal and external group, respectively. None of above parameters was statistically different between two groups.

**Table 2:** Surgical procedure between internal and external intestinal plications

	Internal intestinal plication (n = 9)	External intestinal plication (n = 11)	*p*
Emergent surgery (n, %)	3 (33.3 %)	4 (36.4 %)	0.99
Duration of surgery (min)	240.0 (83.0)	310.0 (115.0)	0.33
Intra-op bleeding (ml)	200.0 (100.0)	300.0 (600.0)	0.11
Intra-op transfusion (n, %)	0	1 (9.1 %)	0.99
Bowel resection (n, %)	6 (66.6 %)	5 (45.5 %)	0.41
Bowel resection (cm)	20.5 (32.5)	0 (13.5)	0.51

### Short-term outcome

There was only 1 patient suffered from surgical site infection in internal group, and none of patients in internal group required reoperation (Table 3). In contrast, there were 6 cases of complications including 3 intra-abdominal bleeding, 2 anastomosis leakage and another surgical site infection in external group. Among these 6 cases, 3 received reoperations including 2 re-laparotomy and 1 DSA (digital substraction angiography) embolization due to postoperative complications. The incidence of postoperative complication was significantly lower in internal group (*p* = 0.043), while re-operation rate was similar between two groups. Notably, 1 patient in external group died from postoperative anastomosis leakage followed by sepsis and MODS.

**Table 3:** Short-term outcome between patients receiving internal or external intestinal plication

	Internal intestinal plication (n = 9)	External intestinal plication (n = 11)	*p*
Post-op complication (n, %)	1 (11.1 %)	6 (54.5 %)	0.043
Intra-abdominal bleeding	0	3 (27.3 %)	–
Anastomosis leakage	0	2 (18.2 %)	–
Surgical site infection	1 (11.1 %)	1 (9.1 %)	–
Perioperative reoperation (n, %)	0	3 (27.3 %)	0.22
Re-laparotomy	0	2 (18.2 %)	–
DSA embolization	0	1 (9.1 %)	–
Post-op ICU stay (day)	0 (0)	0 (1.5)	0.13
Post-op liquid diet restore (day)	8.0 (1.0)	9.0 (16.2)	0.084
Post-op hospitalization (day)	12.0 (2.0)	14.0 (28.5)	0.049
Perioperative death (n, %)	0	1 (9.1 %)	0.99

Postoperative ICU stay (0.22 vs. 1.36 d) and liquid diet restore (7.7 vs. 15.2 d) was shorter in internal group, but failed to reach statistical difference. Postoperative hospitalization was significantly shorter in internal group compared to that in external group (12.1 vs. 25.9 d, *p* = 0.049).

### Long-term outcome

The mean duration of follow-up period in internal and external group was 72.4 and 55.0 months, respectively (Table 4). Two patients in each group presented recurrent bowel obstruction during follow-up period. The readmission rate was low in both groups (22.2 % vs. 9.1 %), and none of patients required reoperation or deceased after discharge.

**Table 4:** Long-term outcome between patients receiving internal or external intestinal plication

	Internal intestinal plication (n = 9)	External intestinal plication (n = 11)	*p*
Duration of follow-up (month)	75.0 (21.0)	57.0 (37.2)	0.18
Recurrent bowel obstruction (n, %)	2 (22.2 %)	2 (18.2 %)	0.99
Readmission (n, %)	2 (22.2 %)	1 (9.1 %)	0.57
Reoperation (n, %)	0	0	0.99
Death during follow-up (n, %)	0	0	0.99

**Fig. 2:**
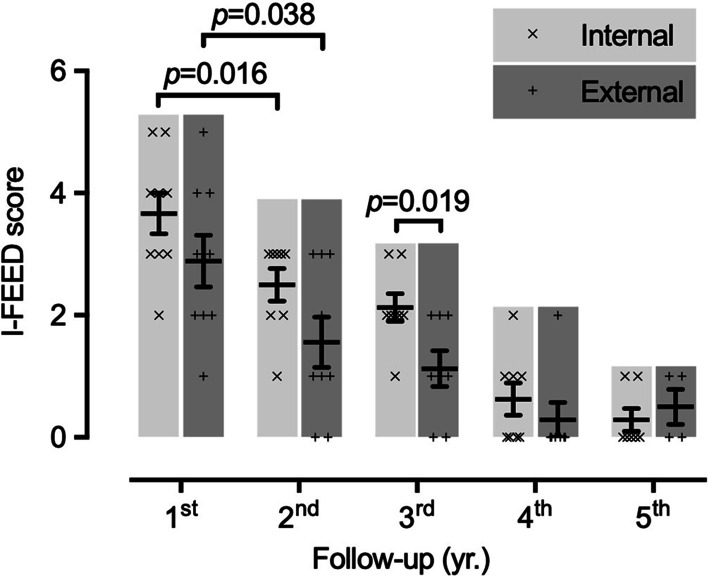
Gastrointestinal function after intestinal plication surgery during 5-year follow-up period. The gastrointestinal function after internal or external intestinal plication was evaluated by I-FEED scoring system recommended by American Society for Perioperative Quality Initiative. The I-FEED score was calculated at each year after discharge. Each symbol represented each patient. Bar graph indicated mean ± sem. Unpaired t-test with Welch’s correction was used for statistical analysis. *I-FEED* intake, feeling nauseated, emesis, physical exam, and duration of symptoms

I-FEED score represented the postoperative gastrointestinal function. Figure 2 demonstrated the trend of I-FEED score during annual follow-up visits. None of patients were higher than 5 points after 1 year, indicating none of patients exhibited gut dysfunction. Afterwards, I-FEED score was gradually and significantly decreasing (*p* = 0.016 vs.* p* = 0.038 between 1st and 2nd annual visits of internal and external group, respectively), and dropped below 2 points at 4th year, indicating that all patients had restored normal gut function after 4 years.

In comparison of gut function recovery between two groups, the I-FEED score was consistently lower in external group, especially at 3rd year when a statistical difference was observed (*p* = 0.019), suggesting an accelerated recovery of gut function after receiving external intestinal plication.

## Discussion

Herein, we summarized our main findings. In short-term perioperative period, the complication rate of external plication reached as high as 54.5 %, including intra-abdominal bleeding, anastomosis leakage and surgical site infection, which was associated with high risk of reoperation and even death. In long-term follow-up period, the complication rate declined dramatically, and none of patients required reoperation. Gut dysfunction disappeared after 1 year, improved during each visit, and eventually returned to normal after 3 years.

The main novelty of our study includes the comparison of outcome between two types of intestinal plication and the long-term follow-up survey of 5 years on average, which has been rarely achieved by previous studies. During the short-term perioperative period, external plication carries more damages and delayed recovery compared to internal plication, which was defined by higher incidence of complication and longer stay of hospitalization. In contrast, external plication is associated with lower I-FEED score at each follow-up visit, indicating an enhanced recovery of gut function after external instead of internal plication. This finding suggests that internal plication might be more suitable for elderly patients or patients with severe malnutrition or under inferior body condition, due to less damage and accelerated recovery during perioperative period. Comparatively, external plication could be prioritized to younger patients or better preoperative condition that could survive from damage of surgery.

Intestinal plication carries considerable damage, and leaves rare chance of reoperation. Considering the high rate of perioperative complication in patients receiving external plication, it is critical to determine the necessity of plication in patients, especially young patients. However, consensus has not been reached regarding the surgical indications for intestinal plication procedure. Previous literature recommends several indications including (1) extensive intra-abdominal/inter-intestinal adhesion that leads to recurrent bowel obstruction; (2) extensive serosa defect that leads to high risk of postoperative adhesive obstruction; (3) abdominal cocoon, intestinal malrotation or volvulus; (4) abdominal trauma, mesentery or intestine severe injury [1, 2, 8, 11, 12].

In this study, the reason for intestinal plication included volvulus, intussusception, abdominal cocoon and adhesive obstruction. Among them, adhesive small bowel obstruction (ASBO) after surgery is the main cause, with an average incidence between 4.8 and 26 % [13, 14]. A bundle of principles including sharp instead of blunt dissection as well as abdominal lavage before closure has been proposed for the prevention of postoperative intra-abdominal adhesion, it is still inevitable in certain cases. Enterolysis is effective for the relief of obstruction but incapable for the prevention of recurrence. Fevang et al. conducted a large study involving 500 patients with SBO and found that the cumulative recurrence rate for ASBO after enterolysis was 18 % after a decade, 29 % after 3 decades and up to 81 % after multiple admissions [15]. Moreover, they identified that the number of abdominal operations was the significant risk factor for ASBO [15]. Therefore, it is necessary to explore a surgical technique for the prevention of ASBO.

In our study, we utilized anterograde splinting of internal plication which we assume is more consistent with anatomy and physiology of human gut. There is also retrograde splinting that has been reported with similar safety and effectiveness to anterograde splinting [2, 11]. Few studies attempted non-surgical intestinal splinting for patients with ASBO [16]. However, the very small size of patients and short-term follow-up period hampers the grade of evidence.

Our study found that intestinal plication was also applicable for intestinal volvulus and intussusception, which has been rarely reported previously. Hochman et al. reported a case experience of external intestinal plication in the treatment of small bowel volvulus subsequent to ileal pouch-anal anastomosis [17]. Altarac et al. reported experimental and clinical outcome of external plication in the treatment of sigmoid volvulus [18]. Aimanan et al. reported their practice of internal splinting in a patient with multiple anastomosis caused by small bowel volvulus [19]. Menzo et al. shared their management experience of intestinal plication in case with intussusception after laparoscopic gastric bypass [20]. Similar practice was also reported in animal studies [21, 22]. All above studies are relatively preliminary. Our data could augment the evidences of intestinal plication in the field of volvulus and intussusception.

Abdominal cocoon is a rare cause of bowel obstruction, most of which require surgical treatment. Enterolysis is not adequate since adhesion and subsequent obstruction would probably occur. Intestinal plication is therefore suggested in addition to enterolysis for the management of abdominal cocoon [23–25]. Nevertheless, the majority of these studies were single-arm studies in the absence of control group, and none of these studies compared external versus internal plication. Our data provided a preliminary insight into this field.

We are aware of our potential limitations. First, this is a retrospective analysis that might bring selection bias. Our data demonstrated matched baseline characteristics between two groups that helps to ensure the reliability of subsequent analysis. Nevertheless, prospective randomized trials are expected in the future. Second, since intestinal plication was considered as a prudent strategy for a specific spectrum of diseases, the sample size is inevitably small in a single hospital. Multi-center large studies are necessary in the future. Third, there lacks comparison of quality of life (QoL) after intestinal plication. Although gut function was inspected at each annual visit, QoL questionnaire was not included in the study. Future studies towards the QoL in patients receiving intestinal plication would assist to complement the outcome after this procedure.

## Conclusions

In conclusion, our study compared short-term and long-term outcome of patients receiving internal or external intestinal plication, which could be used for the management of small bowel obstruction cause by specific diseases including volvulus, intussusception, adhesive obstruction and abdominal cocoon. Considering the high rate of perioperative complication in external group, we suggested a conservative attitude toward external plication strategy. Surgical indication for intestinal plication is critical and awaits future investigations.

## Supplementary Information

**Additional file 1: Table S1.** Overview of patients receiving intestinal plication. 

## Data Availability

The datasets used and/or analysed during the current study are available from the corresponding author on reasonable request.

## Abbreviations

WBC: White blood cell count
CRP: C-reactive protein
