# Supplementary material for: Comparative short-term and long-term outcomes between internal and external intestinal plication in the management of small bowel obstruction
Source: BMC Surg. 2021 Jul 12;21:309. doi: 10.1186/s12893-021-01304-1 (PMC8276395; doi:10.1186/s12893-021-01304-1)
Supplement: Supplementary file 1 — Additional file 1: Table S1. Overview of patients receiving intestinal plication. [file 12893_2021_1304_MOESM1_ESM.docx]

**Table S1. Overview of patients receiving intestinal plication**

|  | Sex | Age | BMI | Primary Dis | Pre-op WBC (×10^9^/L) | Pre-op Hb (g/L) | Pre-op Alb (g/L) | Pre-op CRP (mg/L) | Duration Surg (min) | Intra-op bleeding (ml) | Bowel Resection (cm) | Follow-up (month) |
| --- | --- | --- | --- | --- | --- | --- | --- | --- | --- | --- | --- | --- |
| Internal Plication (n=9) | M | 57 | 23.7 | AC | 5.0 | 132 | 35.9 | 58.8 | 390 | 100 | 0 | 75 |
|  | F | 15 | 17.2 | V | 20.8 | 125 | 35.5 | 102.0 | 240 | 50 | 18 | 17 |
|  | M | 84 | 20.0 | AO | 7.9 | 108 | 29.5 | 6.2 | 217 | 200 | 0 | 73 |
|  | F | 30 | 19.3 | AO | 5.5 | 80 | 28.0 | 0.2 | 180 | 200 | 0 | 97 |
|  | M | 36 | 20.0 | V | 3.2 | 108 | 39.2 | 4.1 | 260 | 200 | 20.5 | 87 |
|  | M | 16 | 19.2 | AO | 3.7 | 135 | 45.3 | 0.2 | 240 | 100 | 34 | 111 |
|  | M | 58 | 20.9 | V | 12 | 112 | 27.6 | 128.4 | 210 | 300 | 47 | 78 |
|  | M | 68 | 20.0 | AO | 5.1 | 123 | 33.3 | 3.2 | 300 | 300 | 32.5 | 48 |
|  | M | 35 | 21.9 | AO | 4.9 | 112 | 44.0 | 1.0 | 335 | 200 | 32 | 66 |
| External Plication (n=11) | F | 67 | 22.8 | AC | 4.6 | 115 | 38.8 | 31.4 | 345 | 300 | 0 | 38 |
|  | M | 76 | 16.0 | AO | 6.3 | 123 | 36.1 | 6.8 | 310 | 100 | 0 | - |
|  | M | 48 | 20.4 | I | 5.5 | 143 | 39.6 | 4.5 | 390 | 1000 | 49 | 59 |
|  | M | 54 | 21.6 | AC | 10.5 | 139 | 33.7 | 42.0 | 300 | 300 | 0 | 28 |
|  | M | 73 | 21.9 | AO | 17.8 | 125 | 33.3 | 118.8 | 240 | 100 | 12 | 88 |
|  | M | 82 | 20.2 | AC | 6.1 | 121 | 21.7 | 161.0 | 320 | 600 | 15 | 83 |
|  | F | 53 | 26.6 | AO | 3.0 | 112 | 36.2 | 4.8 | 660 | 2400 | 37 | 1 |
|  | M | 43 | 19.1 | AC | 6.4 | 134 | 39.1 | 2.7 | 210 | 200 | 12 | 84 |
|  | M | 60 | 19.1 | V | 5.9 | 142 | 42.8 | 2.9 | 180 | 100 | 0 | 64 |
|  | F | 65 | 24.6 | V | 3.5 | 126 | 35.8 | 2.7 | 160 | 100 | 0 | 55 |
|  | F | 22 | 21.3 | AO | 7.8 | 135 | 38.3 | 25.0 | 335 | 800 | 0 | 50 |

*Abbreviations:* AC, abdominal cocoon; AO, adhesive obstruction; V, volvulus; I, intussusception.
